# Exploration of Chlorophyll *a* Fluorescence and Plant Gas Exchange Parameters as Indicators of Drought Tolerance in Perennial Ryegrass

**DOI:** 10.3390/s19122736

**Published:** 2019-06-18

**Authors:** Piotr Dąbrowski, Aneta H. Baczewska-Dąbrowska, Hazem M. Kalaji, Vasilij Goltsev, Momchil Paunov, Marcin Rapacz, Magdalena Wójcik-Jagła, Bogumiła Pawluśkiewicz, Wojciech Bąba, Marian Brestic

**Affiliations:** 1 Department of Environmental Improvement, Faculty of Civil and Environmental Engineering, Warsaw University of Life Sciences-SGGW, 02-776 Warsaw, Poland; piotr_dabrowski@sggw.pl (P.D.); bogumila_pawluskiewicz@sggw.pl (B.P.); 2 Polish Academy of Sciences Botanical Garden-Center for Biological Diversity Conservation in Powsin, 02-973 Warsaw, Poland; 3 Department of Plant Physiology, Faculty of Agriculture and Biology, Warsaw University of Life Sciences-SGGW, 02-776 Warsaw, Poland; hazem@kalaji.pl; 4 Department of Biophysics and Radiobiology, Sofia University “St. Kl. Ohridski”, 1164 Sofia, Bulgaria; goltsev.v@uni-sofia.bg (V.G.); m_paunov@uni-sofia.bg (M.P.); 5 University of Agriculture in Kraków, Faculty of Agriculture and Economics, Department of Plant Physiology, 30-239 Krakow, Poland; rrrapacz@cyf-kr.edu.pl (M.R.); magdalena.wojcik-jagla@urk.edu.pl (M.W.-J.); 6 Department of Plant Ecology, Institute of Botany, Faculty of Biology, Jagiellonian University, 30-387 Krakow, Poland; wojciech.baba@uj.edu.pl or wojciech.baba12@gmail.com; 7 Department of Plant Physiology, Slovak University of Agriculture in Nitra, 949 76 Nitra, Slovakia; marian.brestic@uniag.sk

**Keywords:** plant stress, chlorophyll, turf grass varieties, gas exchange, delayed fluorescence, MR 820

## Abstract

Perennial ryegrass (*Lolium perenne* L.) belongs to the common cultivated grass species in Central and Western Europe. Despite being considered to be susceptible to drought, it is frequently used for forming the turf in urban green areas. In such areas, the water deficit in soil is recognized as one of the most important environmental factors, which can limit plant growth. The basic aim of this work was to explore the mechanisms standing behind the changes in the photosynthetic apparatus performance of two perennial ryegrass turf varieties grown under drought stress using comprehensive *in vivo* chlorophyll fluorescence signal analyses and plant gas exchange measurements. Drought was applied after eight weeks of sowing by controlling the humidity of the roots ground medium at the levels of 30, 50, and 70% of the field water capacity. Measurements were carried out at four times: 0, 120, and 240 h after drought application and after recovery (refilling water to 70%). We found that the difference between the two tested varieties’ response resulted from a particular re-reduction of P700^+^ (reaction certer of PSI) that was caused by slower electron donation from P680. The difference in the rate of electron flow from Photosystem II (PSII) to PSI was also detected. The application of the combined tools (plants’ photosynthetic efficiency analysis and plant gas exchange measurements) allowed exploring and explaining the specific variety response to drought stress.

## 1. Introduction

Urban green areas fulfill numerous functions, the more important being: the reduction of pollutants by phytoremediation [1,2,3] and the regulation of the urban microclimate [4]. On that basis, the concept of green infrastructure was introduced. The basic purpose of that idea was to sustain the high complexity and dynamism of urban areas. Green infrastructure can be outlined as a network of natural and semi-natural areas, where combinations of different functions (ecological, social, and economic) coexist as a whole at different spatial scales, from urban centers to peri-urban areas. The turf, besides the trees and shrubs, is one of the most important parts of green infrastructure. When kept in good condition, it can improve the aesthetic value of the whole city. 

Developing of urban areas is a main cause of the extensive transformation and alteration of land cover, biogeochemical cycles, air quality, and climate. Moreover, the environmental changes at the local scale may even have a stronger impact on vegetation than the global changes. The urban environment is characterized by changes in the surface energy balance, the high concentration of gases, pollutants, and particulate matter (PM) or rainfall patterns, and the promotion of the urban heat island effect [5,6]. These features of urban areas may enhance the stress pressure on the vegetation’s functionality in the form of abiotic and biotic factors, especially water stress [7]. 

Drought considerably affects plant productivity, which is connected mainly with the reduction of photosynthetic activity. That phenomena may be driven by both closing stomata and complex non-stomatal effects, which are not fully recognized nor understood [8]. The changes in Photosystem II (PSII) activity observed during drought may be the effects of both limitations and the effects dependent on the degrees and on the species. Under water deficit, inhibition of PSII activity has been proven for some agricultural species, e.g., wheat [9], barley [10], trees [11], and also *Lolium*/*Festuca* grasses [12,13,14]. On the other hand, the detailed information about the simultaneous drought effect on Photosystem I (PSI) and PSII activity in perennial ryegrass turf varieties is missing. 

Perennial ryegrass (*Lolium perenne* L.) is the grass species of common use for turf in urban areas in all of Central and Western Europe; it is also used as a forage crop [15]. Since the advent of turf varieties in the 1960s, the development of perennial ryegrass varieties has increased steadily. In 2017, more than 400 turf varieties of that species were registered in the EU [16]. 

Many authors have stated that chlorophyll *a* fluorescence (ChlF) parameters are useful for the characterization of the responses of plants to a variety of environmental stress factors and for phenotyping [17,18,19,20]. On the basis of the ChlF technique, it is possible to measure the plant vigor under negative environmental conditions. The measured parameters of that phenomenon are strictly correlated with the PSII functioning [17,18,21]. Green plants under stress conditions change their photosynthetic metabolism. These changes may be direct or indirect, but they are usually correlated with the changes in the yield of ChlF. On the basis of the fluorescence quenching analyses, it is possible to deliver the information about the energy absorption, the utilization, the dissipation, and the electron transport in PSII. 

The photosynthetic function of the plants under stress conditions can be estimated by the analysis of the polyphasic fast chlorophyll transient. That quick and nondestructive method (which is also called prompt fluorescence (PF)) was developed by Strasser and Strasser [22]. The photosynthetic apparatus’ photochemical efficiency is reflected by fluorescence kinetics, and it delivers valuable data about the functional and structural features of the mechanisms engaged in photosynthetic electron transport [23,24]. The fluorescence rises during the first second of illumination from the initial (F_o_) to the maximal (F_m_) value of fluorescence and is shown as a curve with several phases (labeled as O, K, J, I, P) [25,26,27]. 

The PSII antenna complex can also reemit the light absorbed for photochemical reactions, such as delayed fluorescence (DF). However, there are essential differences between PF and DF. The PF signal can be generated by a particular molecule, whereas DF depends on system relations [28,29,30,31,32]. All the redox reactions of the photosynthetic electron transport between PSII and PSI, as well as the total electron transfer reactions in the reaction centers (RC) of PSII (donor and acceptor side) are revocable. The agglomeration of electrons in the electron transport chain between PSI and PSII brings the electron back, and charge recombination in PSII RC occurs. Subsequently, the RC is re-excitated, and fast energy transfer from it repopulates the excited chlorophyll state of the PSII antenna [33].

The modulated light reflection (MR) signal measured at 820 nm delivers information about electron transport after the plastoquinone (PQ) and to the PSI acceptors [33,34]. Based on that, it is possible to detect the influence of stress factor on the redox state of the PSI RC and plastocyanin (PC). 

In the opinion of Goltsev et al. [28], the parallel analyses of the PF, DF, and MR 820 are essential for obtaining the comprehensive relation of the impact of stress factors on electron transport chain (ETC). Because of this correlation, it is possible to reveal the importance of such simultaneous analyses, when adding substance donating or accepting electrons at characteristic locations in the ETC [30]. 

Regarding the hypothesis that the grasses from various places of origin have different adaptive responses to ecological conditions and drought stress may impair multiple sites of the photosynthetic electron chain, we wanted to know: (i) how the photosynthetic apparatus of two perennial ryegrass varieties responds to drought and which mechanisms of the photosynthetic processes are involved, (ii) if there are differences in response of PSII, PSI, and gas exchange between the tested varieties, and (iii) whether the recovery capacity of photosynthetic performance after re-watering can be estimated by chlorophyll fluorescence measurements. Therefore, the detailed *in vivo* analysis and the comparisons of the changes in PSI and PSII photochemistry induced by drought stress in two turf varieties of perennial ryegrass by means of the parameters derived from the prompt chlorophyll fluorescence and delayed chlorophyll fluorescence records and the MR 820 signal were the main aims of this study. We believe that our work expands the knowledge about the differences between these two varieties in the photosynthetic response to drought stress and provides clues to elucidate the possible photosynthetic mechanism by which perennial ryegrass tolerates drought stress. Broadening our knowledge about the impact of the altered response of plants to the fluctuating environment will help to improve predictions of the effect of future climate change on ecosystems and plant communities in urban areas.

## 2. Materials and Methods

### 2.1. Plants, Growth Conditions, and Experiment Design

Two turf varieties of perennial ryegrass (*Lolium perenne* L.), “Roadrunner” (Turf Seed, Inc., Gervais, OR, USA) and “Nira” (Małopolska Hodowla Roślin, Kraków, Poland), were the objects of the experiment. These twos varieties are one of the most commonly used for creating lawns in urban areas in Central Europe. The experiment was conducted in the greenhouse of Warsaw University of Life Sciences (SGGW), under natural lighting conditions (day/night, with a 16-hour day length). The average temperature was equal to 22 °C. The seeds (0.47 g/pot) of both varieties were sown on 11 March 2016 in 13 × 13 × 13 cm pots filled with a mixture of sand (70%) and clay (30%). The single dose of multi-component fertilizer Substral 100 (30 g·m^−2^) was applied. The drought stress was applied 8 weeks after sowing by smaller water dosing (compared to the control). The plants lost water through natural evapotranspiration. Water was dispensed to maintain a homogeneous field water content (FWC) of the substrate. Three treatments of water content were applied: 70% (control), 50%, and 30%. The soil moisture was controlled daily by use of ProCheck needle probe (Decagon Devices, Inc., Pullman, WA, USA). The experiment was set in a split-plot system, where the first treatment was varieties, and the second was level of drought. The whole experiment consisted of 36 pots.

All of the measurements were carried out in uniform conditions on flag leaves in 6 repetitions. The measurements were made at 4 time points. The first time point was performed before drying the soil. The next two time points were conducted 120 and 240 h after the stress application. The last was conducted 160 h after re-watering to 70%. 

### 2.2. The Chlorophyll Fluorescence and the Gas Exchange Measurements

Chlorophyll fluorescence measurements were conducted on 8-week-old plants. Six leaves were dark adapted for 30 min before measurements started. The M-PEA Chlorophyll Fluorescence Measurements System (Multi-Function Plant Efficiency Analyzer, Hansatech, Pentney, UK) was used. The following measurement protocol was applied: measurement time 1.0 s, intensity of actinic light 3000 μmol·m^−2^·s^−1^, wavelength 635 ± 10 nm. The following chlorophyll fluorescence signals were measured: prompt chlorophyll *a* fluorescence, delayed chlorophyll *a* fluorescence, and 820-nm light reflection.

The JIP-test, described by Strasser et al. [33], was used to recalculate the characteristic points of photoinduced chlorophyll fluorescence transients to specific parameters of the light phase of photosynthesis. According to Goltsev et al. [35], the characteristic points (I_1_, I_2_, and D_2_) of the DF curve were also assessed. The I_1_ point is the first maximum of the curve, the I_2_ point the second maximum, and D_2_ the second minimum of the curve. Two ratios were calculated: (I_1_^−^D_2_)/D_2_ and I_1_/I_2_. The MR signal was measured simultaneously with DF. The ratio MR_t_/MR_0_, where MR_t_ is the modulated 820-nm reflection intensity at time t and MR_0_ is the value of the 820-nm reflection of the sample at the onset of the actinic illumination, was calculated. The minimum (MR_min_) and maximum (MR_max_) levels were also calculated, as well as _Δ_MR_fast_ (difference between MR_0_ and MR_min_) and _Δ_MR_slow_ (difference between MR_max_ and MR_min_).

Plants’ gas exchange in terms of net CO_2_ assimilation (*A*), transpiration rate (*E*), stomatal conductance (*g_s_*), and sub-stomatal CO_2_ concentration (*C_i_*), were measured by a portable gas analyzer Lcpro+ (ADC, Bioscentific ltd, Hoddesdon, U.K.). That open-gas exchange system was operated during the measurement in differential mode at a 150-mmol·s^−1^ flow rate of ambient air. The air temperature during measurements was 22 °C. Saturating irradiance was about 1000 μmol·m^−2^·s^−1^. Air relative humidity was 60%. The measurements were made on 6 leaves in each treatment, always after the stabilization of conditions in the chamber. The descriptions of fluorescence parameters, DF, MR820, and gas exchange parameters are described in Table 1.

### 2.3. Statistical Analysis

Both chlorophyll fluorescence and gas exchange parameters were statistically analyzed by the two-way ANOVA model, where the first factor was the level of field water capacity and the second was the variety. The Fischer’s least significant differences test was used as a post hoc test at a 0.05 confidence level. The Statistica 10.0 program (Statsoft, Inc. Tulsa, OK, USA) was used to perform the statistical analysis. The relationship between PF, DF, MR 820 signal, and CO_2_ assimilation was assessed on the basis of the Pearson correlation coefficient. The values of the coefficients were considered only when significant at *p* = 0.05.

## 3. Results

### 3.1. Prompt Chlorophyll a Fluorescence

**Figure 1:**
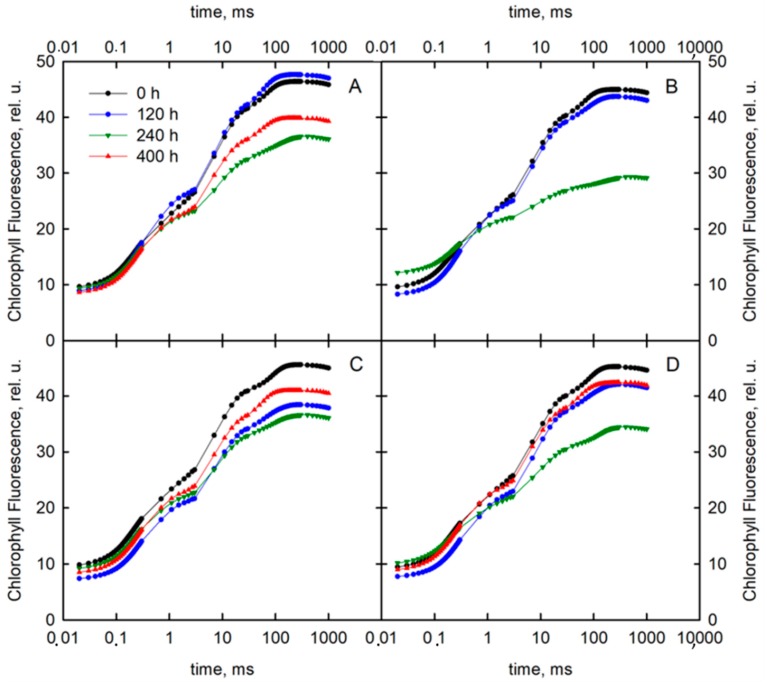
Induction curves of chlorophyll *a* fluorescence of perennial ryegrass varieties (Roadrunner and Nira) grown under different field water capacities of soil (50 and 30% field water content (FWC)) at 0, 120, 240, and 400 h after stress application. (**A**) Roadrunner under 50% FWC, (**B**) Roadrunner under 30% FWC, (**C**) Nira under 50% FWC, (**D**) Nira under 30% FWC. n = 6 relative units. rel. u., relative units.

Water shortage significantly influenced the fluorescent transients in both varieties, especially at the I and P steps of the chlorophyll fluorescence induction curve (Figure 1). In the case of the “Roadrunner” variety grown under control conditions for more than 120 h, the fluorescence signal measured at the I step was at the level of 40 relative units (rel. u.), but after 240 h of 50% FWC stress application, it decreased significantly (by 25%). At this step, re-watering the plants to obtain 70% FWC caused an increase of the signal at this step to 35 rel. u. The signal at the I step after 240 h in plants growing on substrate with 30% FWC was at the level of 26 rel. u., but after this time, it was impossible to take measurements due a severe damage of the plants under that treatment. Similarly, we also were not able to make any measurements of plants’ gas exchange under these conditions. The P step measured in control plants was at the level 45 rel. u.; however, after 240 h of 50% FWC treatment, it was significantly lowered by 26.7%, and under 30% FWC, it was significantly reduced by 33.4%, as compared to control plants. Re-watering plants (raising FWC from 50% to 70%) caused a slight increase of the signals at this step. In the case of the “Nira: variety growing before the stress application, the I step was at the level of 40 rel. u., but after 240 h of 50% FWC treatment, it decreased significantly (by 25%). The I step after 240 h in plants growing on substrate with 30% was decreased by 35%. The P step measured in control plants was at the level 45 rel. u., but after 240 h of stress application, this value decreased significantly by 26% and 22.3% under 50% and 30% FWC conditions, respectively. The re-watering caused an increase in this value in both treatments, but not to the level of control plants. 

**Figure 2:**
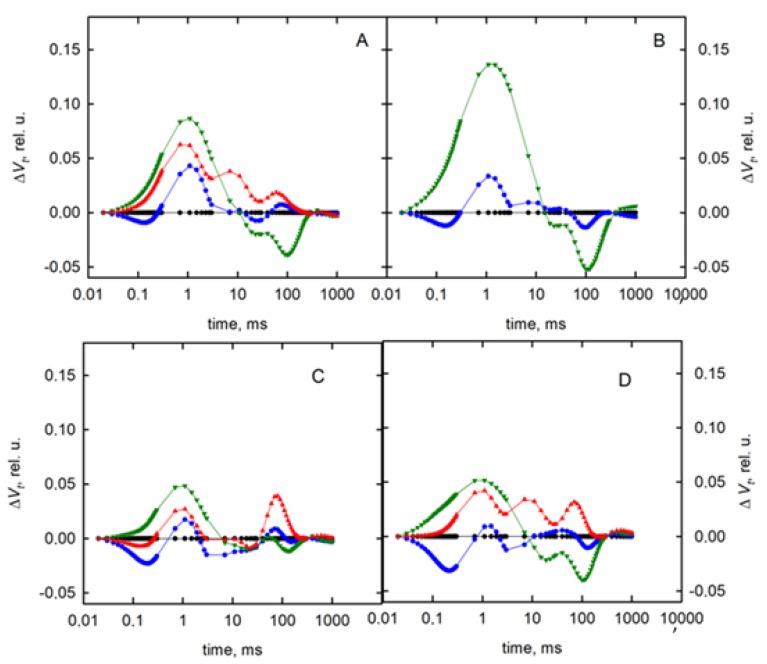
Differential curves of Δ*V_t_* (obtained by subtracting the control curve from the first sample) of perennial ryegrass varieties (Roadrunner and Nira) under different field water capacities of soil (50 and 30% FWC) and times (0, 120, 240, and 400 h after stress application): (**A**) Roadrunner under 50% FWC, (**B**) Roadrunner under 30% FWC, (**C**) Nira under 50% FWC, (**D**) Nira under 30% FWC. n = 6 relative units.

To visualize the influence of drought stress on the dynamics of the chlorophyll fluorescence induction curve, the relative variable fluorescence, V_t_ = (F_t_ − F_O_)/(F_M_ − F_O_), was calculated. In the next step, the calculations of the differences in relative variable fluorescence curves were made by subtracting the normalized fluorescence values (between O and P steps) recorded in control plants under drought stress (ΔV = V_t_(Xh) − V_t_(0h)) [33]. The character of the OJIP fluorescence transients recorded in plants under stress of both varieties differed from those recorded in non-stressed plants (Figure 2). The major changes in the curves’ course of stressed plants were detected in the O–J phase and I–P phase. In the O–J phase, the curve drawn for stressed plants had a higher course than for the control plants, but in the I–P phase, these curves had a lower course. There were also significant differences between the tested varieties.

For complete analysis of drought-induced changes in the OJIP curve, the differential curves were presented separately for main bands occurring during O-P transients: K-, L-, H-, and G-bands. The curves for these bands were constructed by subtracting the normalized fluorescence values (between O and K, O and J, J and P, or I and P, respectively) recorded in control plants from those recorded in plants under drought stress (Appendix A). The appearance of the K-band and L-peak in the fluorescence transients of stressed plants further pointed out the differences between tested varieties in terms of their response to the appearance of stress. 

The case of L-band was quite different in comparison with the K-band for both varieties. The most visible changes occurred not 240 h, but 120 h after stress application. It should be noted that for that variety treated by 30% FWC, only during one treatment, the L-band was at a higher level than in the control plants.

The H-band was the parameter significantly decreased by the stress factor after 240 h. However, a significant decrease was also observed after 120 h. 

The G-band parameter was significantly decreased by the stress application only after 240 h. 

**Figure 3:**
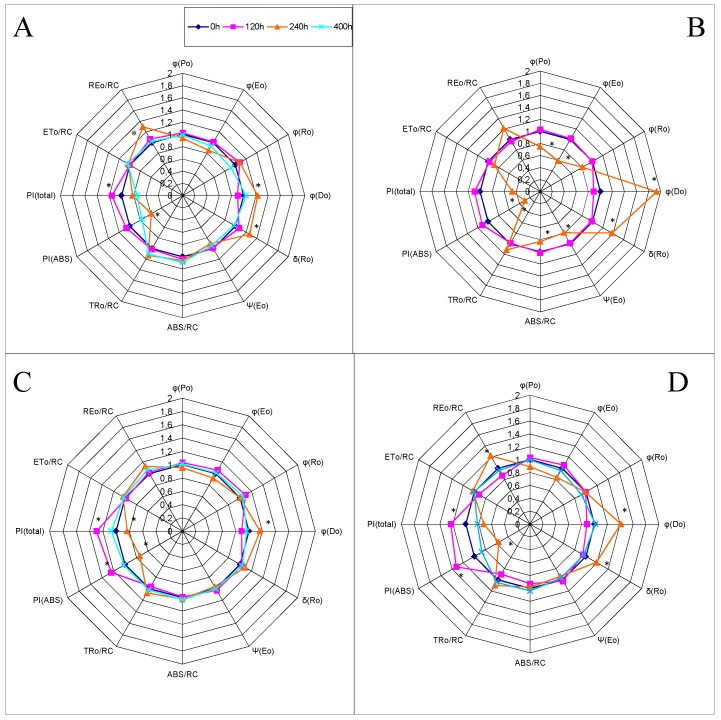
JIP-test parameters normalized to the values before stress application (0 h) as radar plots under different field water capacities of soil (50 and 30% FWC). (**A**) Roadrunner under 50% FWC, (**B**) Roadrunner under 30% FWC, (**C**) Nira under 50% FWC, (**D**) Nira under 30% FWC. Relative units. Means of one parameter marked by an asterisk differ significantly (*p* < 0.05, n = 6).

The OJIP transients were also transformed to the biophysical parameters [33]: the quantum yields (φ_Po_, φ_Eo_, φ_Ro_, φ_Do_, Ψ_Eo_, δ_Ro_), specific activities per reaction center (RC), and performance indices (PI). The values of the calculated parameters were normalized to those of the plants in the control (non-stress) conditions (0 h). The deviations of the action patterns of the stressed and control plants are shown as radar plots (Figure 3). The decrease in field water capacity caused significant differences in some parameters of quantum yields for both varieties. However, the changeable parameters depended on the variety. For the Roadrunner variety, the φ_Do_ and δ_Ro_ parameters were changed under drought stress. Moreover, these changes were greater in plants treated with 30% FWC, than ones treated with 50% FWC. In the case of the Nira variety, only the φ_Do_ parameter was significantly increased by both drought treatments, but once again, the changes were greater in the plants treated with 30% FWC than in plants treated with 50% FWC. Regardless of the variety and the degree of drought, the re-watering caused practically full recovery of the parameters to the control values.

The performance index PI_ABS_ was used to quantify the PSII performance, and the performance index PI_total_ was used to measure the performance up to the reduction of PSI end electron acceptors. The performance indexes were also significantly modified by drought stress in both varieties; decreases in these parameters were observed. For the Roadrunner variety under 30% FWC, both parameters decreased significantly in comparison to others. Regardless of the variety and the degree of drought, the re-watering caused partial recovery of these parameters to the same values as in control plants.

Drought stress caused significant changes in the parameter classified as the specific activity of the reduction of end electron acceptors of PSI per reaction center (REo/RC). In both varieties, the values of REo/RC already increased significantly in the plants growing under 30% FWC. Moreover, the changes were also significant for the Roadrunner variety under 50% FWC. 

### 3.2. Delayed Chlorophyll a Fluorescence

**Figure 4:**
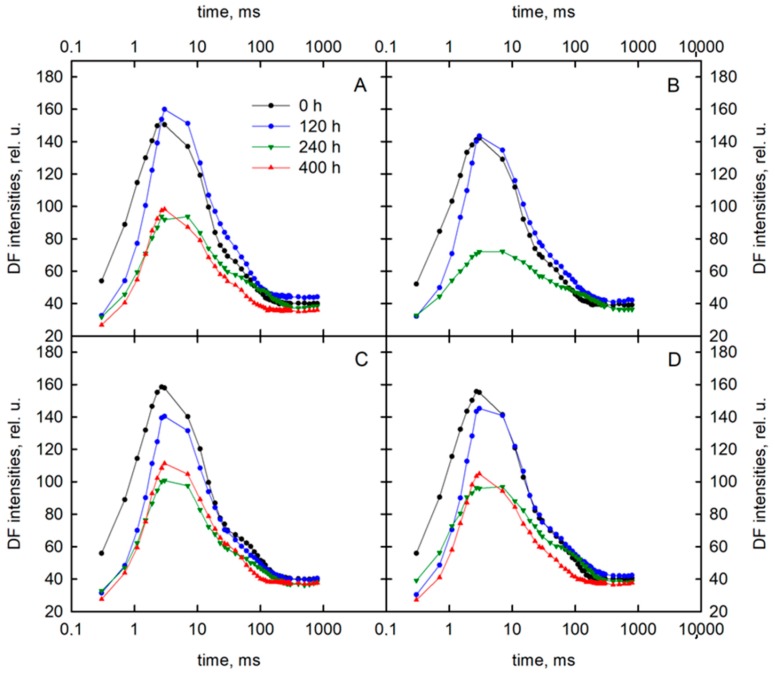
Delayed fluorescence induction curves of perennial ryegrass varieties (Roadrunner and Nira) under different field water capacities of soil (50 and 30% FWC) and times (0, 120, 240, and 400 h after stress application): (**A**) Roadrunner under 50% FWC, (**B**) Roadrunner under 30% FWC, (**C**) Nira under 50% FWC, (**D**) Nira under 30% FWC. n = 6. Relative units.

In this work, we focused on the first recorded delayed fluorescence (DF) decay component that appeared within a microsecond time range. In the initial part of the DF induction kinetics (during the first 1 s of illumination), that emission was the principal share. The changes of the delayed fluorescence induction curves depended on the field water capacity and the variety. The DF values diminished with increasing the time of exposure to stress for both varieties (Figure 4). For the Roadrunner variety growing under 50% FWC, a noticeable decrease in the intensity of curves after 240 h of stress application was observed. At that time point, the first maximum of the curve (I_1_) decreased from 154 rel. u. to 94 rel. u. For the re-watering plants, the curve had a similar course as after 240 h. In this variety treated with 30% FWC, the visible changes were also obtained 240 h after the application of stress, decreasing maximally from 142 rel. u. to 72 rel. u. For the Nira variety treated with 50% FWC, the first changes in the course of the curve were noted 120 h after stress application; at this time, the I_1_ maximum decreased from 161 rel. u. to 140 rel. u. Even greater changes were recorded after 240 h from the stress application. The value of the I_1_ parameter decreased to 101 rel. u. The re-watering caused an increase in this parameter to 111 rel. u. For the plants treated with 30% FWC, the changes were observed only 240 h after stress application, when the maximum of the curve decreased from 166 rel. u. to 97 rel. The re-watering did not cause any visible changes in the values of the maximum. It should be noted that there were no significant differences between the minimums of the curve (D_2_) measured in the control and drought-stressed plants. 

**Figure 5:**
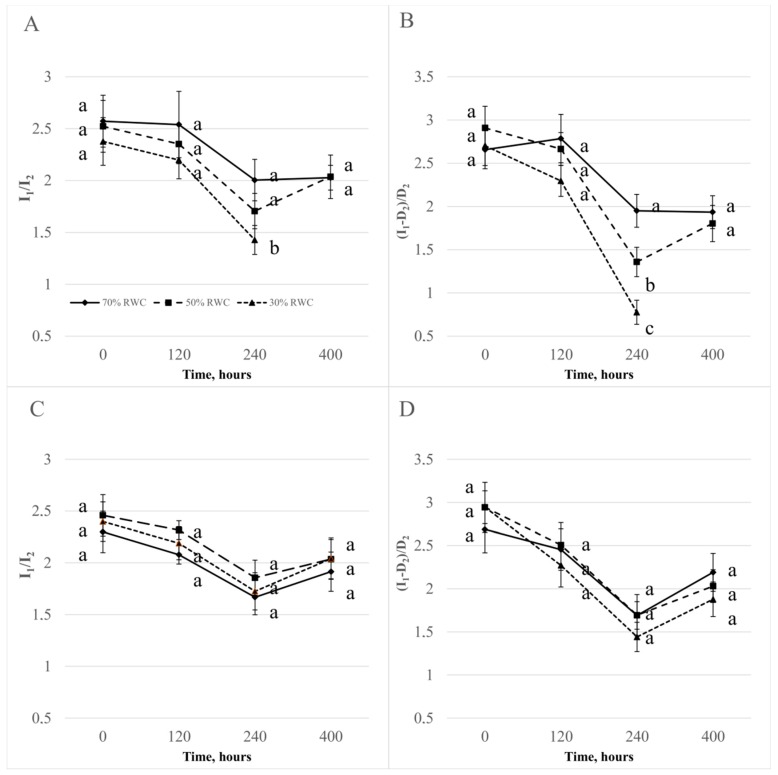
(I_1_-D_2_)/D_2_ and I_1_/I_2_ ratios ± S.D. of perennial ryegrass varieties (Roadrunner and Nira) under different field water capacities of soil (50 and 30% FWC) and times (0, 120, 240, and 400 h after stress application): (**A**) Roadrunner under 50% FWC, (**B**) Roadrunner under 30% FWC, (**C**) Nira under 50% FWC, (**D**) Nira under 30% FWC. n = 6. Relative units. Mean values within a term marked by the same letter did not show significant differences (*p* < 0.05, n = 6).

To better demonstrate the changed shape of the DF, the ratios between the maxima and minima of the induction curves were determined (Figure 5). The ratio I_1_/I_2_ in plants treated with 50% and 30% FWC changed only for the Roadrunner variety after 120 and 240 h. The re-watering from 50%–70% FWC restored the I_1_/I_2_ to the value for control plants. The course of the changes of the ratio (I_1_ − D_2_)/D_2_ was similar to the changes of the ratio I_1_/I_2_. However, for this ratio, the changes were more visible and occurred also for the Nira variety. 

### 3.3. The Modulated Light Reflection Signal Measured at 820 nm

**Figure 6:**
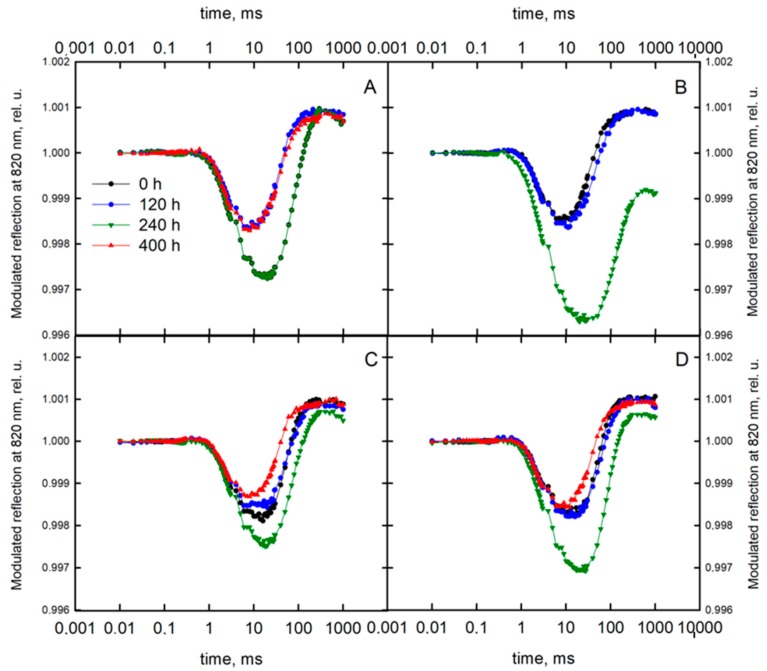
Kinetics of modulated light reflection at 820 nm (all data are normalized to the initial measured value of the signal) of perennial ryegrass varieties (Roadrunner and Nira) under different field water capacities of soil (50 and 30% FWC) and times (0, 120, 240, and 400 h after stress application): (**A**) Roadrunner under 50% FWC, (**B**) Roadrunner under 30% FWC, (**C**) Nira under 50% FWC, (**D**) Nira under 30% FWC. n = 6 relative units.

The reflection at 820 nm was significantly affected by drought stress in both varieties (Figure 6). In both varieties, the values of the MR_min_ parameter were the lowest 240 h after the application of stress in plants treated with both field water capacities. However, the most significant decrease was noted for the Roadrunner variety under 30% FWC. The re-watering increased the course of the curve to a similar level as in control plants in all treatments. The MR_max_ parameter decreased significantly 240 h after applying drought stress in plants treated only with 30% FWC. For the Roadrunner variety, the decrease in that parameter was more pronounced. For the Nira variety, the re-watering caused alignment to the values similar to that of the control plants.

**Figure 7:**
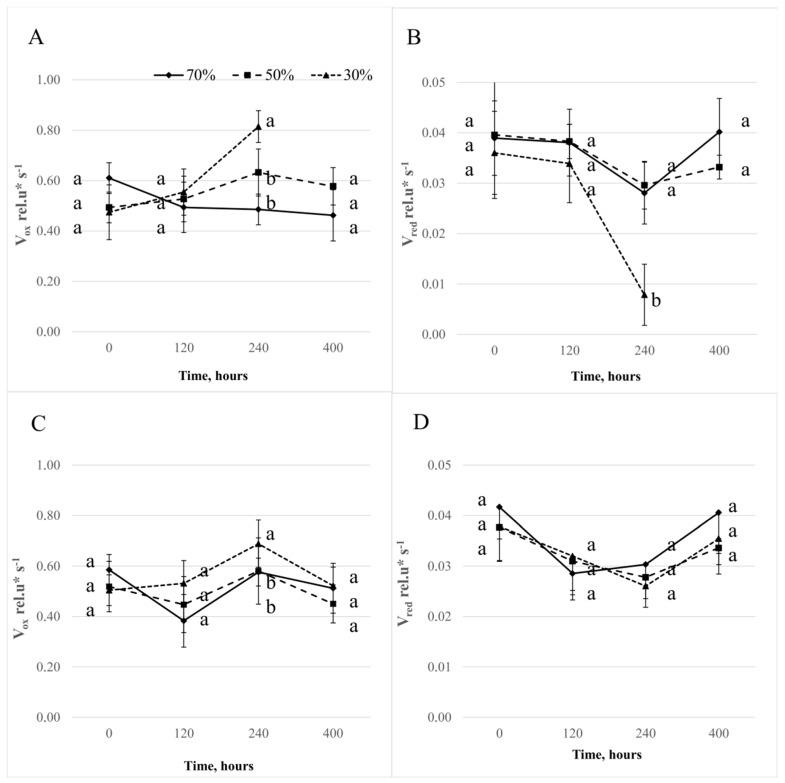
Relative rates of reaction centers of PS I (P700) oxidation (**A**,**C**) and P700^+^ re-reduction (**B**,**D**) ± S.D. calculated from the MR820 signal of Roadrunner (A,B) and Nira (C,D) under different field water capacities of soil (50 and 30% FWC) and times (0, 120, 240, and 400 h after stress application). Relative units. Mean values within a term marked by the same letter did not show significant differences (*p* < 0.05, n = 6).

The rate of initial photooxidation of reaction center of PS I (P700) (V_ox_) for the Roadrunner variety treated with drought conditions increased in the comparison with control plants 240 h after the application of stress (Figure 7). However, the re-watering did not cause any changes: there were still significant differences between the treatments. In the case of the Nira variety, the most visible changes were recorded 120 h after the stress application, but they were less noticeable than for the Roadrunner variety. Moreover, the re-watering caused alignments to the values similar to those of the control plants: the rate of re-reduction of P700^+^ by electrons from PS II and the reduced PQ pool (V_red._). For the Roadrunner variety treated with 30% FWC, this was decreased in comparison to control plants 240 h after stress application. In the case of the Nira variety, the drought treatment did not cause any changes of this parameter. 

### 3.4. Gas Exchange Analyses

**Figure 8:**
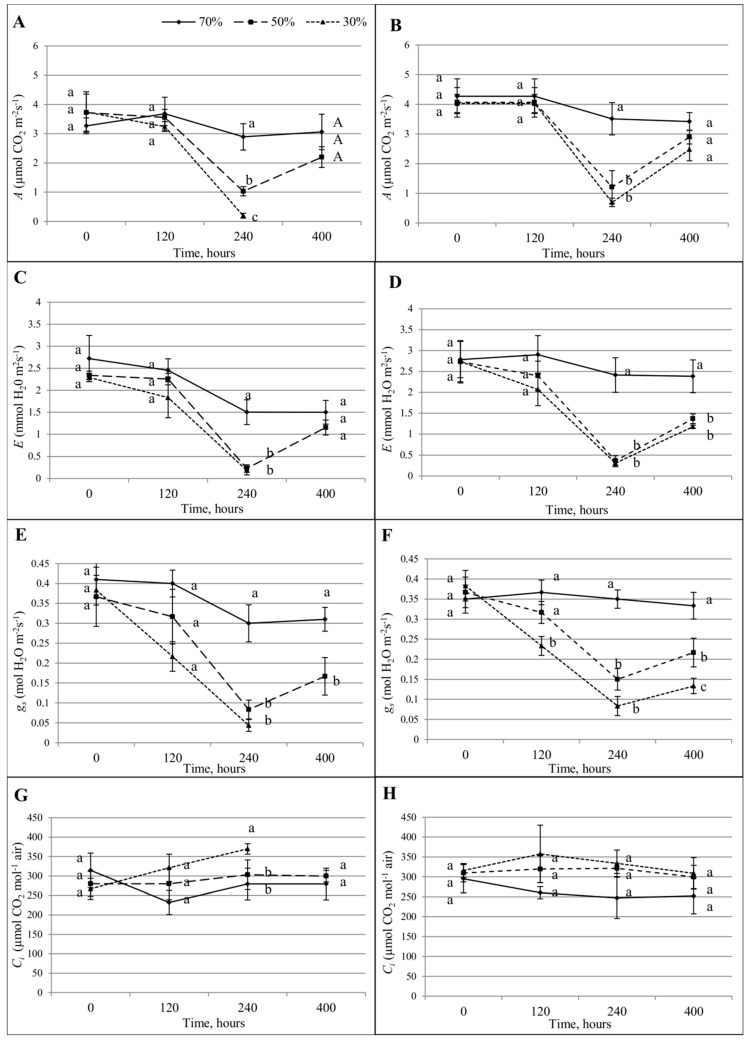
Gas exchange parameters ± S.D. of Roadrunner and Nira under different field water capacities (70, 50, and 30% FWC) and times after the application of stress (0, 120, 240, and 400 h). (**A**) CO_2_ assimilation (*A*) in Roadrunner; (**B**) CO_2_ assimilation (*A*) in Nira; (**C**) H_2_O transpiration (*E*) in Roadrunner; (**D**) H_2_O transpiration (*E*) in Nira; (**E**) stomatal conductance (*g_s_*) in Roadrunner; (**F**) stomatal conductance (*G_s_*) in Nira; (**G**) internal CO_2_ concentration (*C_i_*) in Roadrunner; (**H**) internal CO_2_ concentration (*C_i_*) in Nira. Mean values within a term marked by the same letter did not show significant differences (*p* < 0.05, n = 6).

Plants’ CO_2_ assimilation (*A*) was significantly influenced by drought in the case of both varieties (Figure 8). In the case of the Roadrunner variety, the significant differences between the values measured for all three treatments were detected 240 h after stress application. The CO_2_ assimilation measured in the control plants was equal to 3.2 µmol CO_2_ m^−2^·s^−1^ and decreased by 65% for the plants under 50% FWC and by 95% for the plants under 30% FWC. The re-watering from 50%–70% FWC caused the value of that parameter to increase to similar values as for the control plants. In the case of the Nira variety, significant differences were also observed 240 h after stress application. However, there were no significant differences between plants treated with 50 and 30% FWC. The CO_2_ assimilation measured in the control plants was equal to 4.2 µmol CO_2_ m^−2^·s^−1^ and decreased by 73% and 80% respectively in plants under drought stress. Re-watering from 50% and 30%–70% FWC increased the value of the parameter to a similar level as in the control plants.

Plants’ transpiration rate € in the case of the Roadrunner variety was affected significantly 240 h after the stress application. In the control plants, that parameter was equal to 1.5 mmol H_2_O m^−2^·s^−1^ and decreased to 0.4 mmol H_2_O m^−2^·s^−1^ for the plants treated with both levels of FWC. The re-watering from 50%–70% caused recovery of that parameter to similar values as for the control plants. In the case of the Nira variety, the transpiration was affected significantly also 240 h after the application of stress. The value measured on the control plants was at a level of 2.4 mmol H_2_O m^−2^·s^−1^. Drought stress caused a decrease in the *E*-value to 0.4 mmol H_2_O m^−2^·s^−1^ (83% in the comparison to the control) in the plants treated with both levels of FWC. The re-watering from 50% and 30%–70% FWC increased the values of that parameter, but they were still lower in comparison to control plants.

Stomatal conductance (*g_s_*) was also affected significantly by the drought stress. For the Roadrunner variety, a significant decrease was noted 240 h after stress application. The value measured for the control was equal to 0.30 mol H_2_O m^−2^·s^−1^. The value measured in the plants treated with the 50% FWC decreased by 67% and by 83% for the 30% FWC treatment when compared to the control. The values measured in treatments with both levels of FWC comprised one homogenous group. The re-watering caused an increase in the values of this parameter, but they were still lower than for the control plants. In the case of the Nira variety, the significant differences were noted 120 h after stress application. The value measured for control plants was equal to 0.36 m^−2^·s^−1^ and decreased by 34% in plants treated with 30% FWC. At the next time points (240 h), the value measured in the control plants was equal to 0.35 mol H_2_O m^−2^·s^−1^, decreasing by 57% for the plants treated with 50% FWC, and by 74% for the plants treated with 30% FWC. The significance of the differences between the 50% and 30% treatments was found. The re-watering from 50% and 30%–70% FWC caused an increase in the values of that parameter, but they were still lower in comparison to the control plants.

The internal CO_2_ concentration (*C_i_*) for the Roadrunner variety was significantly influenced 240 h after the stress application. For the control plants, that parameter was at the level of 270 (µmol CO_2_ mol^−1^ air) and was higher only for the plants treated with 30% FWC (331 µmol CO_2_ mol^−1^ air). In the case of the Nira variety, the value of this parameter measured in control plants ranged from 250–300 (μmol CO_2_ mol^−1^ air), and there were no significant changes between them and the plants treated by drought. 

### 3.5. Relationships among ChlF Parameters and Gas Exchange

**Figure 9:**
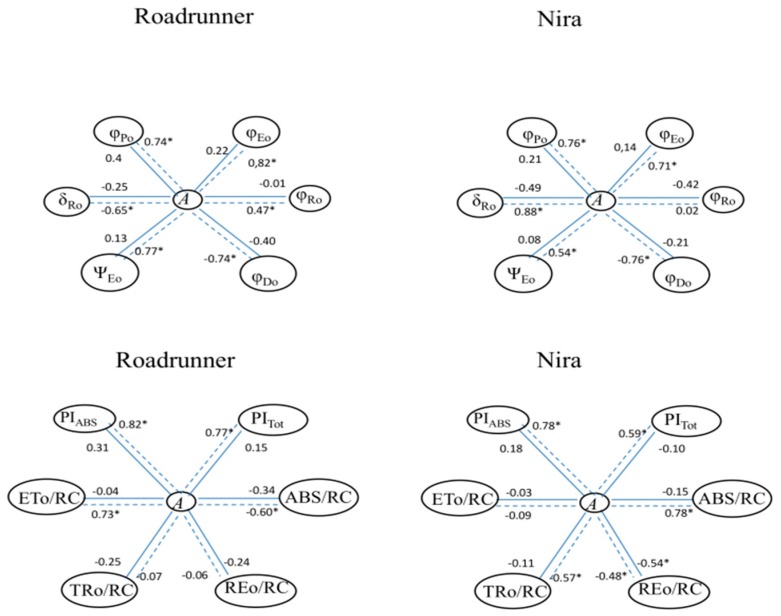
Pearson correlation coefficients (*r*) between prompt fluorescence parameters and CO_2_ assimilation (*A*) (correlations significant at *p* < 0.05, n = 6). Relations between parameters marked by a solid line were calculated for plants in control conditions, and those marked by a dotted line were calculated for plants under 30% FWC.

On the basis of the statistical analysis, it can be noted that there were significant correlations between some PF parameters and the CO_2_ assimilation parameter (*A*) (Figure 9). First of all, the significant correlations were noted in both varieties only in stress conditions. Under drought stress in the Roadrunner variety, significant correlations were noted in the majority of the JIP test parameters. Only REo/RC and TRo/RC were not correlated with the *A* parameter. In the Nira variety also, only two parameters were not correlated with A: φRo and ETo/RC.

**Figure 10:**
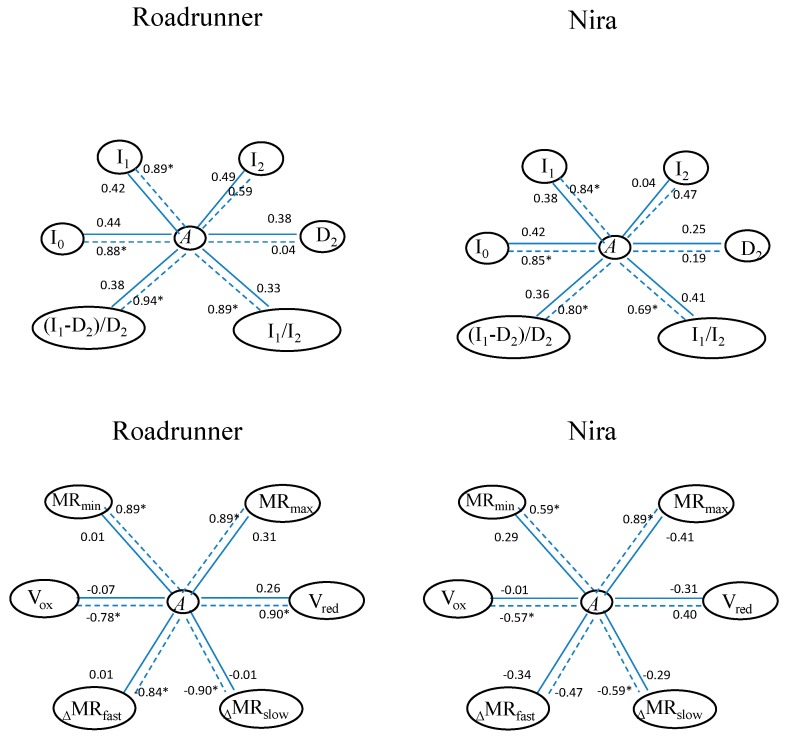
Pearson correlation coefficients (*r*) between delayed fluorescence parameters and CO_2_ assimilation (*A*) and between modulated reflection at 820 nm and CO_2_ assimilation (*A*) (correlations significant at *p* < 0.05, n = 6). Relations between parameters marked by a solid line were calculated for plants in control conditions, and those marked by a dotted line were calculated for plants under 30% FWC.

On the basis of the statistical analysis, it can be noted that there were significant correlations between some DF parameters and the CO_2_ assimilation parameter (Figure 10). For the Roadrunner variety in control conditions, there were no significant correlations between the *A* and all DF parameters, but in the plants treated with 30% FWC, *A* was significantly correlated with almost all DF parameters, and the correlation coefficient ranged from 0.84–0.94. Only the parameters I_2_ and D_2_ were not correlated with *A*. A similar phenomenon was noticed in the case of the Nira variety: *A* in the plants treated with drought conditions was significant correlated with all parameters (r ranged from 0.69–0.84), except I_2_ and D_2._ The statistical analysis showed also the significant correlations between the *A* parameter and the MR curve parameters in both varieties treated with drought conditions. There were no significant correlations in control plants. For the Roadrunner variety, the parameters MR_min_, MR_max_, V_red_, and _Δ_MR_fast_ were significant and positively correlated with the *A* parameter (r ranged from 0.84–0.90), while the parameters _Δ_MR_slow_ and V_ox_ were correlated negatively with the *A* parameter (r ranged from −0.90 to −0.78). In the Nira variety, the parameters MR_min_ and MR_max_ were correlated positively (r ranged from 0.59–0.80), while the parameters _Δ_MR_slow_ and V_ox_ were correlated negatively (r ranged from −0.59 to −0.57).

## 4. Discussion

Drought is one of the main factors that inhibits plant functioning [36]. Excessive drought brings increased osmotic stress, promptly [37]. Despite numerous studies conducted to understand the mechanism by which drought stress affects plant photosynthesis, this topic is still under investigation [38,39]. Generally, factors that limit photosynthesis under drought stress can be divided into stomatal or nonstomatal ones. However, in most study cases, both effects seem to act simultaneously [40]. Reduction of the *A* parameter is attributed to stomatal limitation if C_i_ decreases and *g*_s_ increases with the decrease in soil FWC. If not, the reduction of *A* is attributed to nonstomatal limitation [41]. However, such reactions are dependent on the studied genotype, the growth stage of the plant, and the level of drought stress.

Ohashi et al. [42] showed that, under drought stress, stomatal closure contributes to maintaining the high leaf water content; however, it leads to a decrease in leaf photosynthesis. Our results proved that under drought stress, the photosynthesis (*A*) and the transpiration (*E*) decreased. Similar phenomena were noticed for stomatal conductance (*g_s_*). The photosynthesis intensity of stressed plants recovered to a value similar to that of plants grown under control conditions after re-watering the soil. This proves that both perennial ryegrass varieties were able to repair metabolic processes within a short time, with the particular case of electron transfer between the photosystems. Chlorophyll *a* fluorescence parameters measured after re-watering reached values similar to those measured for control plants (nearly complete reactivation of photosynthetic processes occurred). These results are in agreement with those obtained by Pukacki and Kamińska-Rożek [43].

The mechanism limiting photosynthesis under drought stress is complex, especially when drought reaches a serious level of severity. Our study showed that the analysis of the leaf gas exchange parameters alone was insufficient to expose the extent of injury in the photosynthetic apparatus caused by drought completely. Additional analyses related to nonstomatal limitation of photosynthesis (based on chlorophyll fluorescence measurements), which can contribute to the reduced activity in the PSII and PSI, helped us to obtain better understanding on the mechanisms of plant tolerance to drought [39].

Measurements of chlorophyll *a* fluorescence kinetics deliver exhaustive information on the structure and function of the photosynthetic apparatus, in particular PSII [25,44,45]. Thus, drought can directly or indirectly change the kinetics of chlorophyll *a* fluorescence [46,47]. Changes in chlorophyll *a* fluorescence parameters connected with drought response were compared before in drought-tolerant and -susceptible genotypes of *Lolium perenne*, *Festuca arundinacea*, and their hybrids [12,48]. Accompanying proteomic and lipidomic studies revealed differences in thylakoid connected proteins (e.g., ATP-ase, oxygen-evolving enhancer protein), in enzymes linked to light-independent photosynthetic processes (e.g., RuBisCO activase, chloroplast fructose-bisphosphate aldolase), as well as in proteins involved in plastid gene expression [11,12]. Furthermore, considerable changes in both the lipid composition of chloroplast membranes and the abundance of enzymes involved in their metabolism were observed [12,13].

The JIP-test analyses in the cases of *Augea capensis* Thunb. and *Zygophyllum prismatocarpum* E. Meyer ex Sond., from the Namibian Desert, showed that drought caused an increase in the fluorescence of both species under all experimental conditions in comparison to the control plants. The regulation of photochemical activity is carried out primarily through the deactivation of PSII reaction centers. In the opinion of those authors, the PI_ABS_ parameter calculated from the JIP-test was a precise and sensitive pointer of the physiological condition of plants, as well as in the field and under laboratory conditions. There was a high correlation between the decreased CO_2_ assimilation capacity and the decreased PI_ABS_ values, which is a multiparametric expression of three independent parameters: the density of reaction centers, which corresponds to the absorption flux, the quantum yield of trapping, and the probability that a trapped exciton will move an e^−^ into the ETC beyond Q_A_ [49]. That finding provided the evidence of the connection of OJIP fluorescence changes during drought in field or laboratory conditions with changes in the general ability of photosynthesis. The integrated regulation of the entire photosynthesis process takes place in such a way that the internal balance between the efficiency of the photosynthetic light phase reaction and the efficiency of reactions leading to the CO_2_ assimilation is maintained [36]. Our study confirmed that PI_ABS_ is one of the most sensitive parameters amongst JIP-tests for drought stress. 

The decrease in the field water capacity caused significant differences in some parameters for quantum yields in both varieties. However, the parameter that would be changed depends on the variety. For the Roadrunner variety, the φ_Do_ and δ_Ro_ parameters changed heavily under drought stress. In the case of the Nira variety, only the φ_Do_ parameter was significantly reduced by both drought treatments. The quantum yield parameters are known to be accurate as the indicators of abiotic stress factors. That is because they reflect the lower efficiency of the regulation of the excitation energy utilization and dissipation by the photosynthetic membrane. It is also known that minimizing the reduction of quantum yield under stress should be advantageous for the yield [10]. In the opinion of Oukarroum et al. [50], a lower δ_Ro_ level indicated a decreased outflow of electrons at the acceptor side of PSI caused by an inactivation of ferredoxin NADP^+^-reductase. Interestingly, the drought tolerance-dependent differences in ferredoxin NADP^+^ reductase were observed before in *Festuca arundinacea*, a drought-tolerant grass genetically similar to and easy to produce hybrids with *Lolium perenne* [12].

Drought application caused the induction curves for both varieties to be at significantly lower levels in comparison to the control. Similar results were observed by Oukarroum et al. [25] for barley plants subjected to drought stress, albeit mainly for the I–P phase (reduction of electron acceptors), and the variation in fluorescence kinetics resulting from various degrees of tolerance in individual varieties. Drought stress may also change characteristic points of the OJIP curve and reduce fluorescence intensity at the J, I, and P steps [51]. In our study, the L-peak was influenced by increasing FWC, which might be evidence that the stability and structure of PSII RCs, as well as antennae, were affected. At the same time, the occurrence of the K-peak was noted in the OJIP transient, and _Δ_W_K_ rose with extended treatment duration. This phenomenon can be associated with oxygen evolving complex (OEC) inactivation and/or inhibition of electron transport on the donor or acceptor side of PSII [27]. It was also noted that the K-peak increased significantly under drought stress. Because of that, the L- and K-peaks may be advised as potential indicators for physiological instability under drought stress before the appearance of their visible signs. The oxygen-evolving enhancer protein 2 was reported to be differentially accumulated in drought-tolerant and -susceptible genotypes of *Festuca arundinacea* [12]. In our work, differences in the activity of the oxygen evolving complex between the tested cultivars can also be proven by the differences in the H- and G-bands. A higher activity can be expected in the case of the Nira variety. Therefore, this is consistent with the results of the study cited above. In this work, we proved that the DF signal may provide important information about the processes in PSII and the photosynthetic membrane in drought-stressed plants. Nevertheless, such an elaborate model as the JIP-test has not yet been proposed for the DF induction curves. 

Based on our work, it can be concluded that the shape of the DF induction curve was influenced by this stress. This phenomenon was confirmed by Goltsev et al. [35]. From the analysis of DF, further insights can be derived by Strasser et al. [33] and Goltsev et al. [35]. Goltsev et al. [35] proved that during the initial stages of the desiccation of bean plants, the overall intensities of DF were not affected significantly until the RWC reached about 20%. When the water content was decreased below 20%, the intensity of DF decreased. The authors concluded that in a clear contrast to the overall intensity, the shape of the DF induction curve was sensitive to water stress in the initial stages of the drought stress. In the range of RWC between 100 and 50%, the characteristic peak in the DF induction curve (I_2_) disappeared. The authors of that work concluded that from the DF parameters, the I_1_/I_2_ ratio was most sensitive to mild drought stress. A well expressed linear dependence on RWC between 100% and 40% RWC was observed. Interestingly, again, an almost linear dependence, but not with the same slope, was observed at lower RWC: I_1_/I_2_ sharply decreased between 20% and 5% and then, at even lower RWC, again slightly increased.

In particular, I_1_, I_2_, and D_2_ parameters were negatively affected by drought in both tested varieties, indicating an enhanced electron transfer capacity at both the donor side and the acceptor side of PSII; also, re-oxidation of PQH_2_, as well as oxidation of P700, became slower [52]. The reduction of I_2_ and D_2_ probably means that the re-oxidation capacity of PQH_2_, the formation of the proton gradient, and the reducing activity of the PSII complex were reduced. We also demonstrated that in the case of the Roadrunner variety, that water loss from 70%–30% of FWC caused a significant increase in the ratio between the two maximums in the DF induction curves. This is evidence of the high sensitivity of this technique to show the photosynthetic responses that are not detectable by other methods. 

The differences in the drought response between the Roadrunner and the Nira varieties were also demonstrated on the basis of the analysis of the MR parameters. For the Roadrunner variety, a decrease in the V_red_ parameters was observed. A similar trend, but not so pronounced, was observed for the Nira variety. According to Oukarroum et al. [53] and Gao et al. [52], the decrease in that parameter under drought stress was evidence of a slower re-reduction of P700^+^, which is the result of slower electron donation from P680. These authors showed also a simultaneous reduced rate of photo-induced electron transfer through PSI (decrease in V_ox_), which was the result of the reduction of the PSI photochemical activity. Our results did not confirm the decrease of the V_ox_ parameter under drought stress. In the opinion of many authors [34,54], the MR signal is mostly influenced by the rate of electron flow from PSII to PSI. In addition, there is also a possibility that a limitation on the acceptor side of PS I was the reason for the changes in the MR signal. On the basis of our results, it can be concluded that the ΔMR_slow_ and ΔMR_fast_ parameters measured for the drought-stressed plants were significantly changed in comparison to the control. In addition, our results did not confirm the correlation between those parameters and the gas exchange parameters. These results are in opposition to the findings of some other studies. Schansker et al. [34] suggested that these parameters could be affected by stress. These authors concluded that the fast phase of the signal corresponds to the kinetics of the photoinduced changes in the P700 redox state, being significantly changed only by strong stress. At the same time, the slow phase of the MR signal, reflecting a P700^+^ re-reduction, decreased gradually with stress intensity. In the opinion of Goltsev et al. [55] and Salvatori et al. [56], the deactivation of this kinetic phase was demonstrated as the reduction of the electron transport rate through plastoquinone to P700^+^, which is the reason for the more stable fast phase of the MR signal.

Altogether, the simultaneous analyses of the JIP-test, DF, MR, and gas exchange showed that drought stress led to deactivation of PSII RCs, decreasing in the connectivity between independent PSII units, and the limitation of electron transport beyond QA. Moreover, the damage of PSI acceptor-side electron transporters and the split between the activities of PSI and PSII were noted. The results obtained from the four types of simultaneously-measured parameters provided much more detailed information about the drought-induced changes in the photosynthetic electron chain than in the case of the application of any of them, separately. This conclusion was confirmed by Zhou et al. [57] for maize. Moreover, in comparison with the less resistant variety, Roadrunner, the destructive influence of drought stress on the electron transport chain of the more resistant variety Nira showed a lesser extent and appeared earlier. In the case of the resistant Nira variety, the drought stress showed a less obvious negative effect on all sections of the OJIP curve, as well as on the energetic connectivity between PSII and PSI oxidation and reduction. At all those points, changes were pronounced faster in the case of the Roadrunner variety. These results proved that the resistant variety may preserve higher photosynthetic activity under drought stress. Thus, it can be said that the simultaneous measurement of OJIP, DF, and MR may be used to distinguish the tolerant perennial ryegrass varieties from the sensitive ones.

## 5. Conclusions

Analysis of obtained data showed that the reaction of the photosynthetic machinery of perennial ryegrass to drought stress is a very complicated process. On the basis of the simultaneous measurements of PF, DF, MR 820 signals, and plant gas exchange parameters, we were able to observe specific effects of water shortage on the photosynthesis processes. That include the reduction of electron flow rate through PSII and inhibition of electron transfer from the reduced PQ pool to the PSI reaction center (P700^+^) and reoxidation of Q_A_^−^. Moreover, an inhibition of the quantum yields of photoinduced electron transport in PSII reaction centers to Q_A_ together with a reduction of the fast phase of photoinduced kinetics of the MR signal were also detectedunder this stress. Additionally, we found that the combination of such applied in this research methods is potentially useful for the non-destructive estimation of relative water content in leaves. However, due significant differences found between the two tested varieties, it is recommended to extend research to a larger population of perennial ryegrass accessions.

## Figures and Tables

**Table 1:** The description of fluorescence parameters (after Strasser et al. 2010 [33]), delayed fluorescence (DF), MR820, and gas exchange parameters. All fluorescence parameters are in relative units. RC, reaction center.

V_J_ = (F_J_ − F_0_)/(F_M_ − F_0_)	Relative variable fluorescence at the J-step
φ_Po_ = 1 − F_0_/F_M_	Maximum quantum yield of primary photochemistry (at t = 0)
φ_Eo_ = (1 − F_0_/F_M_)(1 − V_J_)	Quantum yield of electron transport (at t = 0)
φ_Ro_ = (1 − F_0_/F_M_)(1 − V_I_)	Quantum yield of reduction of end electron acceptors at the PSI acceptor side (reaction center-RE)
φ_Do_ = F_0_/F_M_	Quantum yield (at t = 0) of energy dissipation
Ψ_Eo_ = 1 − V_J_	Probability (at t = 0) that a trapped exciton moves an electron into the electron transport chain beyond Q _A_^−^
δ_Ro_ = (1 − V_I_)/(1 − V_J_)	Efficiency/probability with which an electron from the intersystem electron carriers moves to reduce end electron acceptors at the PSI acceptor side (RE)
t(F_M_)	Time (in m/s) to reach the maximal fluorescence intensity F_M_
PI_ABS_ = γ_RC_/(1 − γ_RC_) × φ_Po_/(1 − φ_Po_) × Ψ_Eo_/(1 − Ψ_Eo_)	Performance index (potential) for energy conservation from exciton to the reduction of intersystem electron acceptors
PItotal = PI_ABS_ × δ_Ro_/(1 − δ_Ro_)	Performance index (potential) for energy conservation from exciton to the reduction of PSI end acceptors
ABS/RC = (1 − γ_RC_)/γ_RC_	Absorption flux (of antenna Chls) per RC
M_0_	Approximated initial slope (in ms^−1^) of the fluorescence transient V = f(t)
TR_0_/RC = M_0_(1/V_J_)	Trapping flux (leading to Q_A_ reduction) per RC
ET_0_/RC = M_0_(1/V_J_)Ψ_0_	Electron transport flux (further than Q _A_^−^) per RC
RE_0_/RC = M_0_(1/V_J_)(1 − V_I_)	Electron flux reducing end electron acceptors at the PSI acceptor side per RC
DI_0_/RC = (ABS/RC − TR_0_/RC)	Dissipated energy flux per RC (at t = 0)
RC/CS_0_ = φ_Po_ (V_J_/M_0_) F_0_	Density of RCs (Q_A_ reducing PSII reaction centers)
I_1_ and I_2_	Maximum of the DF induction curve
D_2_	Minimum of the DF induction curve
MR_0_	Modulated 820-nm reflection intensity at Time “0”
MR_min_	Minimum of modulated 820-nm reflection intensity
MR_max_	Maximum of modulated 820-nm reflection intensity
_Δ_MR_fast_	Fast phase (oxidation) of reflection intensity = MR_0_ − MR_min_
_Δ_MR_slow_	Slow phase (reduction) of reflection intensity = MR_max_ − MR_min_
*A*	CO_2_ assimilation (µmol CO_2_ m^−2^·s^−1^)
*E*	H_2_0 transpiration rate (mmol H_2_O m^−2^·s^−1^)
*G_s_*	Stomatal conductance (mol H_2_O m^−2^·s^−1^)
*C_i_*	Sub-stomatal CO_2_ concentration (µmol CO_2_ mol^−^^1^ air)

**Figure A1:**
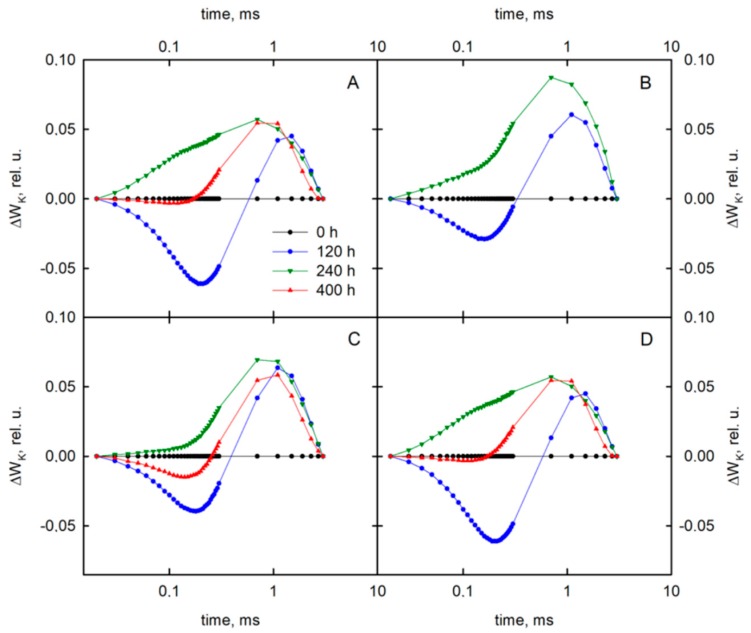
Differential curves of _Δ_W_K_ (obtained by subtracting the control curve from the first sample in the time from 0.02 to 3 ms) of perennial ryegrass varieties (Roadrunner and Nira) under different field water capacity of soil (50 and 30% FWC) and time (0, 120, 240 and 400 h after stress application): (**A**) Roadrunner under 50% FWC, (**B**) Roadrunner under 30% FWC, (**C**) Nira under 50% FWC, (**D**) Nira under 30% FWC. n = 6. Relative units.

**Figure A2:**
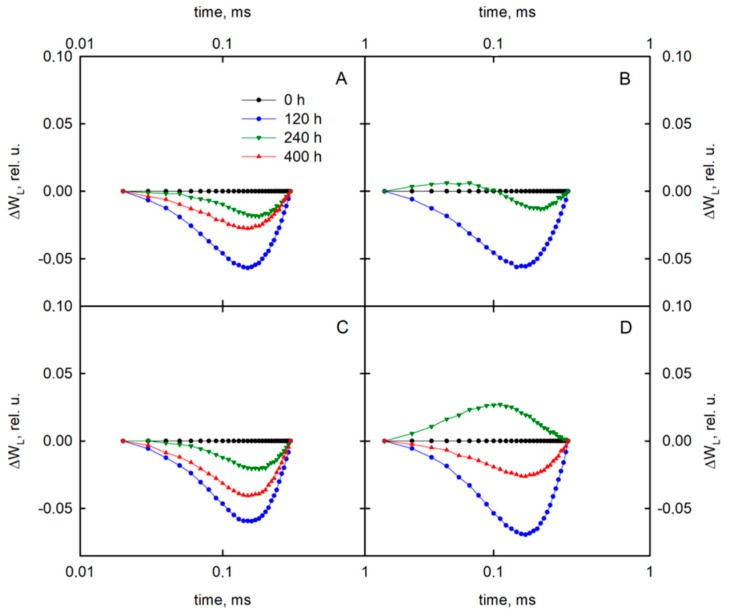
Differential curves of _Δ_W_L_ (obtained by subtracting the control curve from the first sample in time from 0.02 to 0.3 ms) of perennial ryegrass varieties (Roadrunner and Nira) under different field water capacity of soil (50 and 30% FWC) and time (0, 120, 240 and 400 h after stress application): (**A**) Roadrunner under 50% FWC, (**B**) Roadrunner under 30% FWC, (**C**) Nira under 50% FWC, (**D**) Nira under 30% FWC. n = 6. Relative units.

**Figure A3:**
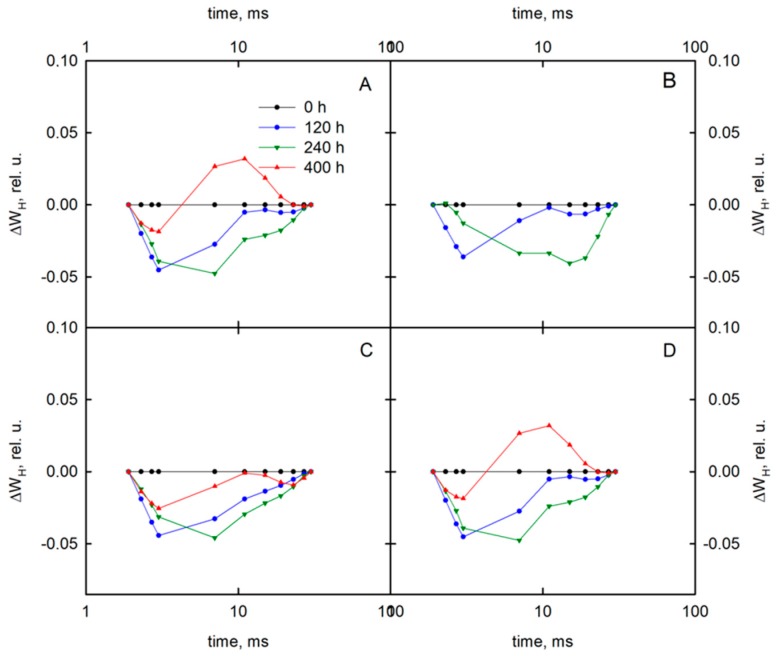
Differential curves of _Δ_W_H_ (obtained by subtracting the control curve from the first sample time from 1.9 to 30 ms) of perennial ryegrass varieties (Roadrunner and Nira) under different field water capacity of soil (50 and 30% FWC) and time (0, 120, 240 and 400 h after stress application): (**A**) Roadrunner under 50% FWC, (**B**) Roadrunner under 30% FWC, (**C**) Nira under 50% FWC, (**D**) Nira under 30% FWC. n = 6. Relative units.

**Figure A4:**
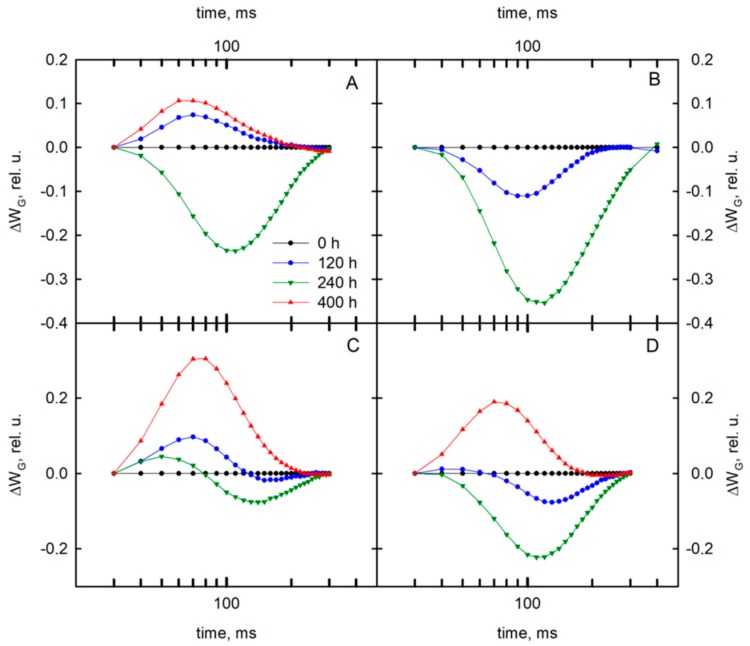
Differential curves of _Δ_W_G_ (obtained by subtracting the control curve from the first sample in time from 30 to 1000 ms) of perennial ryegrass varieties (Roadrunner and Nira) under different field water capacity of soil (50 and 30% FWC) and time (0, 120, 240 and 400 h after stress application): (**A**) Roadrunner under 50% FWC, (**B**) Roadrunner under 30% FWC, (**C**) Nira under 50% FWC, (**D**) Nira under 30% FWC. n = 6. Relative units.
